# Sample size requirements for pilot randomised controlled trials with binary outcomes: a simulation study

**DOI:** 10.1186/1745-6215-14-S1-O21

**Published:** 2013-11-29

**Authors:** Dawn Teare, Munya Dimairo, Alexandra Hayman, Neil Shephard, Amy Whitehead, Stephen Walters

**Affiliations:** 1 University of Sheffield, Sheffield, UK

## Aims

There is lack of consensus over what sample size should be used for pilot trials to inform the design of definitive randomised controlled trials. The majority of the existing recommendations focus on estimating the variation of a continuous outcome and relatively little attention is paid to binary outcomes. The aim of this research was to investigate the sample size required to precisely estimate parameters such as event, consent and attrition rates. We assume that the pilot study (and the definitive RCT) will be a two parallel group treatment versus control design.

## Methods

We simulated binomially distributed outcome data with true event rates between 0.1 to 0.5. We examined the variation in estimates, of the event rate, from pilot study sample sizes of 10 to 150 in incremental steps of 5. We assessed the precision of the estimated event rate by calculating the relative a 95% confidence interval (CI) using the Wilson method, and the percentage gain in precision per increase in sample size of 5.

## Results

The width of the confidence interval plots suggests a sample size that once a sample size of 60 is achieved the relative gain in precision for each additional 5 subjects drops below 5%.

## Conclusions

If the primary outcome of a planned RCT is a binary outcome then the pilot should contain 60 subjects in each group (120 in total) to estimate the event rate with a reasonable degree of precision. This is substantially larger than the currently common practice regarding continuous outcomes.

